# As they wait: Anticipatory neural response to evaluative peer feedback varies by pubertal status and social anxiety

**DOI:** 10.1016/j.dcn.2021.101004

**Published:** 2021-08-12

**Authors:** Selin Topel, Stefon J.R. van Noordt, Cynthia J. Willner, Barbara C. Banz, Jia Wu, Peter Castagna, Elise D. Kortink, Melle J.W. van der Molen, Michael J. Crowley

**Affiliations:** a Developmental and Educational Psychology, Institute of Psychology, Leiden University, the Netherlands; b Clinical Psychology, Institute of Psychology, Leiden University, the Netherlands; c Yale Child Study Center, Yale University, United States; d Department of Psychology, Mount Saint Vincent University, Halifax, Nova Scotia, Canada; e Department of Emergency Medicine, Yale University, United States; f Leiden Institute for Brain and Cognition, Leiden University, the Netherlands

**Keywords:** Adolescence, Social anxiety, Puberty, Anticipation, SPN, Social evaluation

## Abstract

Adolescence is a developmental period characterized by substantial biological, neural, behavioral, and social changes. Learning to navigate the complex social world requires adaptive skills. Although anticipation of social situations can serve an adaptive function, providing opportunity to adjust behavior, socially anxious individuals may engage in maladaptive anticipatory processing. Importantly, elevated social anxiety often coincides with adolescence. This study investigated cortical electroencephalogram (EEG) responses during anticipation of evaluative feedback in 106 healthy adolescents aged 12–17 years. We examined differences in anticipatory event-related potentials (i.e., stimulus preceding negativity [SPN]) in relation to social anxiety levels and pubertal maturation. As expected, the right frontal SPN was more negative during feedback anticipation, particularly for adolescents with higher social anxiety and adolescents who were at a more advanced pubertal stage. Effects for the left posterior SPN were the opposite of those for the right frontal SPN consistent with a dipole. Anticipatory reactivity in adolescence was related to social anxiety symptom severity, especially in females, and pubertal maturation in a social evaluative situation. This study provides evidence for the development of social anticipatory processes in adolescence and potential mechanisms underlying maladaptive anticipation in social anxiety.

## 1 Introduction

Adolescence marks a period of heightened sensitivity to social contexts (Blakemore and Mills, 2014; Guyer et al., 2016; Schriber and Guyer, 2016), and is a common time for the onset of psychiatric conditions, such as social anxiety disorder (Kessler et al., 2005, 2012). Socially anxious adolescents may show disruptions in anticipatory processes, especially when anticipating potential social-evaluative threat (Clark and Wells, 1995; Grupe and Nitschke, 2013; Hinrichsen and Clark, 2003). For example, high socially anxious individuals show heightened anticipatory responses to uncertain social situations and perceive them as more negative (Hofmann, 2007). This could lead to avoidance of social situations and maintaining social impairments over time. Thus, it is critical to understand the processes involved in anticipation of potential social threat, especially during adolescence.

Adolescence is defined as the transitional period between the onset of puberty and adulthood when individuals join new social circles, form romantic relationships and begin to assume more adult-like roles. The adolescent brain goes through a series of neuroanatomical and functional changes thought to promote adaptation to changing social environmental demands (Blakemore and Mills, 2014; Crone and Dahl, 2012; Forbes and Dahl, 2010; Guyer et al., 2016). Neurobiological models of adolescent development (e.g., dual-systems model; Casey et al., 2008; Shulman et al., 2016) attempt to explain this sensitivity and responsivity to social-affective stimuli with the earlier development of subcortical (e.g., limbic) compared to cortical (e.g., prefrontal cortex [PFC]) systems (Schriber and Guyer, 2016). Given the role of the PFC in cognitive control and emotion regulation, it is plausible to think of adolescents’ impulsivity and poor emotion regulation in relation to continuing brain maturation. Hence, difficulty in attaining adaptive emotion regulation skills, as well as increased sensitivity to social stressors, may lead to the emergence of affective problems in some youth.

Given that anxiety is a future-oriented emotion, the uncertain nature of the future can lead to maladaptive responses in highly anxious individuals during anticipation (Grupe and Nitschke, 2013). EEG methods (e.g., ERPs) are well-suited to isolate more automatic aspects of anticipation of social feedback, due to its high temporal resolution. The stimulus-preceding negativity (SPN) has been used to examine the anticipation of different kinds of feedback stimuli (e.g., monetary and social, performance-contingent and gratuitous, visual, auditory, painful) (Ait Oumeziane et al., 2017; Greimel et al., 2018; Masaki et al., 2010; Seidel et al., 2015). The SPN is a negative-going right frontal-dominant ERP component (Brunia et al., 2011; Kotani et al., 2009) with largest amplitude ∼200 ms before the onset of the feedback. The SPN is sensitive to uncertainty and motivational value of the anticipated outcome, such that it is more negative during the anticipation of highly uncertain and motivationally salient stimuli (Ait Oumeziane et al., 2017; Kotani et al., 2017; Mühlberger et al., 2017; Tanovic and Joormann, 2019; Zheng and Liu, 2015), as well as social performance relative to neutral feedback (Ait Oumeziane et al., 2017).

In the only study to date examining the SPN to anticipation of social evaluative feedback, van der Molen et al. (2014) used a social judgment paradigm (SJP) in a sample of 31 young adult females. During the SJP, participants were led to believe that a group of peers evaluated them (i.e., like or dislike), based on first impressions formed after viewing the participant’s picture. Participants were shown pictures of peers and were asked to indicate whether or not they expected to have been liked by that particular peer before they were presented with ‘yes’ or ‘no’ peer feedback. Van der Molen et al. (2014) found that the SPN for anticipated acceptance was larger and more negative than the SPN for anticipated rejection. Moreover, the SPN during anticipation of acceptance was larger among individuals with elevated levels of fear of negative evaluation, a core element of social anxiety. These findings suggest the SPN is useful for studying anticipatory processes relevant to social anxiety. To date, no studies have adopted the SJP to consider the neural correlates of peer feedback anticipation in adolescence, a time in development when maturational changes, pubertal changes and greater incidence of problematic social anxiety converge. The relevance of examining developmental effects on anticipatory processes have been demonstrated by studies employing imaging techniques (Gunther Moor et al., 2010; Hoogendam et al., 2013; Smith et al., 2020) and more recently in an ERP study by Feldmann et al. (2021). These authors reported a decline in SPN amplitudes when anticipating negative outcome valences (e.g., no rewards / punishment) from childhood to late adolescence. A finding that was interpreted to suggest the developmental changes in punishment sensitivity. However, in remains to be tested whether adolescents’ anticipatory EEG responses to social evaluative feedback are associated with similar developmental changes, and how these anticipatory processes relate to pubertal and social anxiety influences.

Our study sought to address the gap in the literature by investigating adolescents’ expectations about social evaluative peer feedback, as well as the neural responses (i.e., the SPN) while adolescents anticipate this feedback. Further, social anxiety and pubertal influences on these feedback predictions and the SPN were examined. The following hypotheses were formulated: (1) We expected to see an overall positive social evaluative feedback expectancy bias (van der Molen et al., 2014, 2018); (2) Based on literature suggesting that socially anxious individuals expect more negative social evaluation, we predicted that adolescents with higher levels of social anxiety would show a negative social evaluative feedback expectancy bias (Caouette et al., 2015; van der Molen et al., 2014); (3) We also expected that high socially anxious adolescents would recall having received fewer acceptance feedback responses, showing a negative memory bias (Glazier and Alden, 2017, 2019; van der Molen et al., 2018); (4) Regarding the SPN, we hypothesized a positive association between SPN amplitudes and social anxiety levels (Spielberg et al., 2015), which would be more pronounced for anticipation of peer acceptance compared with peer rejection (van der Molen et al., 2014). Additionally, we investigated whether the SPN would be larger in older adolescents and adolescents with more advanced pubertal development, since brain development studies have found that self-reported pubertal status may better describe the changes in brain development than age (Wierenga et al., 2018).

## 2 Method

### 2.1 Participants

Initially, our sample consisted of 109^1^ healthy adolescents (51 females) between 12–17 years old (M_age_ = 14.44, SD = 1.72) who participated in a large-scale study employing additional paradigms which are not within the scope of this article, thus not reported here. After excluding one participant due to EEG recording problems and low-quality EEG data, one participant due to large amount of missing demographic and self-report data, and another participant due to insufficient and disproportional number of trials for each condition (133 for expected rejection, 12 for expected acceptance), the final sample of 106 adolescents (M_age_ = 14.44, 50 females) was used for our analyses (see Table 1). All adolescents participating in the study had corrected-to-normal vision and none had a current diagnosis of psychiatric or pervasive developmental disorder or history of traumatic brain injury with loss of consciousness. The majority of the participants were right-handed (i.e., 94.3 %). All the participants were recruited from New Haven, CT and the surrounding towns through a mass mailing list. Among our participants, nine (8.5 %) reported their ethnicity as African American, five (4.7 %) as Asian, six (5.7 %) as Hispanic, and 82 (77.4 %) as Caucasian. Four (3.8 %) participants were reported as having other or unknown racial/ethnic origins. Participants were fluent English speakers. The study was approved by the Human Investigation Committee of the Yale University School of Medicine. Adolescents and their parents/guardians were compensated with $80 USD and $10 USD respectively for their participation in the study. Please see supplementary materials for our full study assessment battery.

**Table 1:** Sample demographics.

Demographic type	Value
Age (mean [SD])	14.9 (1.7)
Age range (min-max)	11.5−17.9
Sex (female/male)	56/50
Handedness (right/left)	100/6

### 2.2 Materials and procedure

#### 2.2.1 Social judgment paradigm

The adapted version of the Social Judgment Paradigm (SJP) was employed in the study (Gunther Moor et al., 2010; Somerville et al., 2006; Van der Molen et al., 2014). A cover story was used in this task where participants were led to believe that they were participating in a study on first impressions. Two weeks prior to the testing day, portrait photographs were collected from the participants. The participants were told that a panel of peers would evaluate them based on their photographs by reporting that the peer either liked or disliked the participant. When participants came into the lab on testing day, approximately two weeks later, they received the instructions that they would be shown the photographs of the peers who evaluated them and were asked to indicate whether they thought each peer liked or disliked them. In reality, the participants were not evaluated by actual peers and the like/dislike feedback was generated by computer. Following the participants’ response, the fictitious peer feedback was presented in each trial in a pseudo-random order. Participants received acceptance feedback 50 % of the time. Different combinations of participants’ expectancies and the feedback they were shown resulted in four experimental conditions upon receipt of feedback: expected acceptance, expected rejection, unexpected acceptance, and unexpected rejection. Given that the current study focused on the period after participants reported their expectations and right before they received feedback, there were two conditions for anticipation: anticipation of acceptance and anticipation of rejection from peers.

**Fig. 1:**
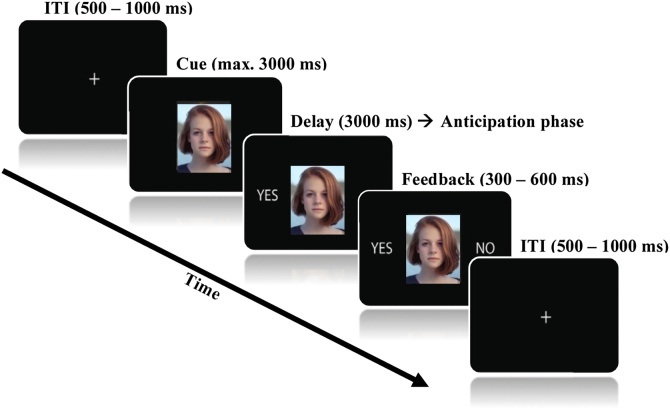
Schematic outline of anexperimental trial. All trials start with a fixation cross (ITI) and end with the offset of the Feedback presentation.

A diverse in-house set of adolescent stimuli collected at the Developmental Electrophysiology Lab was used in the task which consisted of 160 peer photographs with a neutral facial expression (50 % female). Stimuli were presented on a 19-inch monitor with a refresh rate of 60 Hz using E-prime 2.0 software (Psychology Software Tools, Pittsburgh PA). Trial schematic for the experiment is shown in Fig. 1.

In each trial, a photograph of a peer was presented as a cue for a maximum duration of 3000 ms during which the participants were required to provide a response showing their expectancies. If they did not provide their response within this time-interval, they were presented with the feedback “too slow”. For each peer stimulus, participants indicated whether they expected to receive acceptance (“YES”) or rejection (“NO”) feedback from that particular peer by pressing one of the two buttons with their index fingers. The order of which buttons corresponded to acceptance and rejection expectancies was counterbalanced across participants. Following the participants’ response was a delay period for the fixed duration of 3000 ms which was used to study anticipation. The duration of feedback presentation was varied across conditions with the purpose of generating a condition-specific marker for the heart rate recordings which are not within the scope of the current report. Specifically, feedback for the “Yes – Yes” condition (expected acceptance) was presented for 300 ms, feedback for the “Yes – No” (unexpected rejection) was presented for 400 ms; and for “No – Yes” (unexpected acceptance) and “No – No” (expected rejection) the durations were 500 ms and 600 ms respectively. Feedback presentation was followed by a jittered intertrial interval (ITI) between 500–1000 ms where the participants were shown a fixation cross in the middle of the screen. There were 10 practice trials in the beginning of the task preceding the three experimental blocks containing 50 trials each. Mean number of trials used in the analyses was 76.11 (SD = 17.9) for anticipated rejection and 71.33 (SD = 18.36) for anticipated acceptance condition. Following the task administration, participants filled out a number of self-report questionnaires. Debriefing was done through a letter provided after each session was completed.

#### 2.2.2 Self-report questionnaires

##### 2.2.2.1 Social anxiety

We were interested in examining dimensional severity of social anxiety, thus, in line with the recent attempts to quantify the latent variables (Smith et al., 2020), we administered three measures of social anxiety—the Multidimensional Anxiety Scale for Children social anxiety subscale (MASC-SA; March et al., 1997), the Social Phobia and Anxiety Inventory for Children (SPAI-C; Beidel et al., 1995), and the brief version of Fear of Negative Evaluation Scale (bFNE; Carleton et al., 2006)—with the purpose of capturing different aspects of the latent construct of social anxiety.

The MASC is a 39-item (4-point Likert ranging 0–3) scale that measures different anxiety dimensions such as physical symptoms, harm avoidance, social anxiety, and separation anxiety in children and adolescents (March et al., 1997). The social anxiety subscale (MASC-SA) consists of nine items related to humiliation/rejection and performing in public. The SPAI-C measures social anxiety in children through self-reports on a set of 26 items (3-point Likert ranging 0–2) related to assertiveness, social encounters, and public performance (SPAI-C; Beidel et al., 1995). Lastly, the bFNE is a commonly used measure of social anxiety and consists of 12 items (5-point Likert scale ranging 0–5) related to worries about and fears of being negatively evaluated (bFNE; Carleton et al., 2006).

We evaluated the internal consistencies, the mean and the range of the total scores of each self-report questionnaire measuring social anxiety for our sample that are shown in Table 2. The three social anxiety measures were highly positively correlated with each other (Table 3); and they are all measures of a single latent component as indicated by the factor loadings (MASC-SA = .93, SPAIC = .88, bFNE = .85) obtained through a principal component analysis (PCA). Thus, we created a composite social anxiety variable using the component scores generated by the PCA of the MASC-SA, SPAIC, and bFNE measures (Song et al., 2013).

**Table 2:** Mean and range of total scores, internal consistency and number of cases for clinical cutoffs.

Questionnaire	Mean (SD)	Range (min-max)	Internal consistency (Cronbach’s alpha)	Cases > Social anxiety cutoff (cutoff score)^a^
MASC - SA	11.90 (6.14)	0−27	.82	36 (13.5), 22 females
SPAI-C	13.63 (9.95)	0−44.28	.96	30 (18), 19 females
bFNE	17.92 (11.23)	0−48	.94	33 (25), 16 females

a Recommended cutoff scores based on Beidel et al. (1995), Carleton et al. (2011), and Wood et al. (2002).

**Table 3:** Pearson correlations and component loadings of the social anxiety measures.

	MASC-SA	SPAIC	BFNE	Component loadings
MASC-SA	1	–	–	.925
SPAIC	.76**	1	–	.883
BFNE	.69**	.59**	1	.852

*Note.* ** indicates a significance level of *p* < .001.

##### 2.2.2.2 Pubertal development

Adolescents’ pubertal development was measured by the Pubertal Development Scale (PDS), a self-report measure that assesses the physical changes related to puberty in girls and boys (Carskadon and Acebo, 1993). The PDS includes 5 items (4-point Likert scale ranging 1–4) assessing pubertal status based on growth spurt, pubic hair growth, and changed in skin in both boys and girls, in addition to gender-specific changes (e.g., change in voice and facial hair for boys, and breast development and menstrual cycle for girls). Responses of adolescents to all of the items in the PDS were averaged in order to calculate the total scores. The sample mean for the total scores was 3.04 (SD = 0.70) and the scores ranged between 1.3–4.

#### 2.2.3 EEG recording and signal processing

EEG data were recorded using NetStation 4.2 with a high-impedance amplifier (Series 200 Amplifier; Electrical Geodesic Inc., Eugene, OR). Hydrocel-128 channel nets (Electrical Geodesic Inc., Eugene, OR) with conductive gel or saline electrolyte were used for the data collection. All the impedances were below 40 kΩ prior to the recording. The sampling rate was set at 1000 Hz with 0.1–400 Hz frequency band for the recordings, and Cz was used as the online reference. A low-pass filter of 30 Hz was applied to the recorded EEG data. The data were segmented to 3500 ms pre- and 700 ms post-feedback (equivalent to 500 ms pre- and 3700 ms post-response) intervals. Eye blinks and eye movements were both identified based on the eye channel amplitudes that were above 150 μV and corrected using the Ocular Artifact Removal Tool in NetStation 4.5 (Electrical Geodesic Inc., Eugene, OR). Channels that contained data points larger than 200 μV in more than 40 % of their segments were marked as bad channels. Trials that had more than 10 bad channels were identified as bad trials and were excluded from analyses. Spherical spline interpolation was used to replace the bad channels by the surrounding channels. The data were re-referenced offline to the common average reference. Baseline correction was conducted based on a time window of 2400 – 2000 ms before the feedback presentation (as described in Van der Molen et al., 2014). Following the baseline correction, trials for the same conditions (i.e., acceptance and rejection expectancies) were averaged for the further ERP analyses. Among all the participants in the analysis, the acceptance expectancy had a range of 16–108 trials, and the rejection expectancy had a range of 21–100 trials. The mean number of trials for acceptance (*M* = 61.46, *SD* = 18.36) and rejection (*M* = 64.62, *SD* = 17.03) did not differ significantly, *t* (105) = 1.106, *p* = .271.

**Fig. 2:**
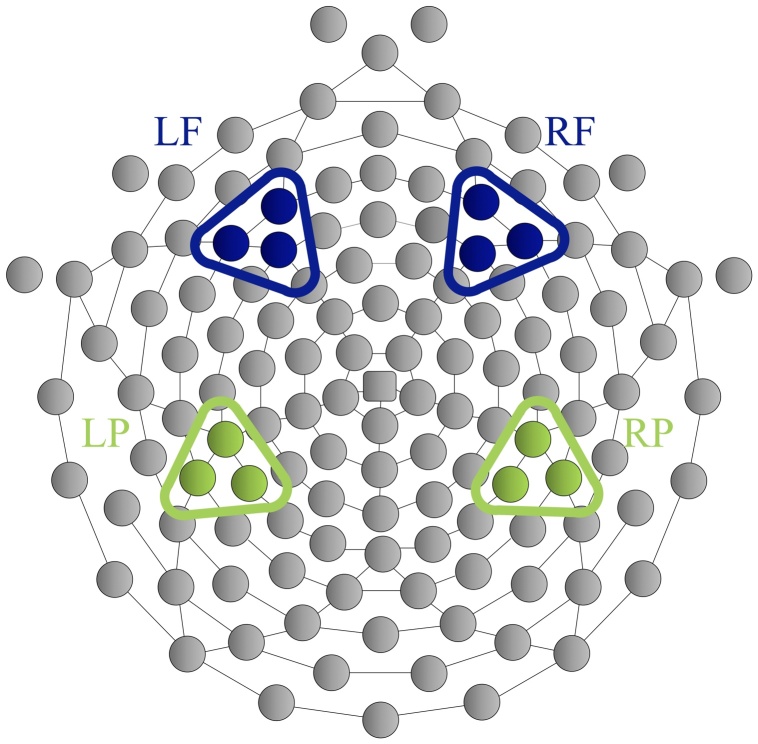
Bilateral frontal and posterior channel clusters used in the analyses. The right frontal cluster (RF) consisted of channels 3, 123, 124 (F4); the left frontal cluster (LF) consisted of 23, 24 (F3), 27. The right posterior cluster (RP) contained channels 92 (P4), 97, 98, and the left posterior cluster (LP) contained channels 47, 51, 52 (P3).

The SPN was calculated by taking the mean amplitude within the 200 ms interval before the feedback presentation (i.e., 2800–3000 ms following the response) at both bilateral frontal and posterior channel clusters (see Supplemental Table S1 and Fig. S1 for reliability and topo plots), considering the mixed findings in the literature regarding the electrode sites where SPN effects were observed (e.g., right anterolateral [F4]; Masaki et al., 2010; Hackley et al., 2020; frontal [Fz, F1, and F2]; Tanovic and Joormann, 2019; left-parietal occipital [PO7]; van der Molen et al., 2014). Fig. 2 shows the channels that right (F4) and left frontal (F3), as well as right (P4) and left posterior (P3) clusters contain on the EGI 128-channel Hydrocel EEG net (Electrical Geodesic Inc., Eugene, OR).

#### 2.2.4 Statistical analyses

All the statistical analyses were conducted using IBM SPSS (version 27). To test whether the adolescents showed an overall positive expectancy bias as indicated by their expectations reported prior to the task and their expectations on each trial throughout the task, separate one-sample *t*-tests were carried out comparing participants’ baseline expectancy to be liked (measured prior to the task) and percentage of their acceptance expectations (measured during the task) to a neutral baseline (50 %). Pearson correlation analyses were performed to test the relationship between baseline and trial-based expectancies to be liked and the level of social anxiety.

We further explored whether the adolescents showed a positive or negative recall bias with a *t*-test comparing how much they remembered to have been liked by peers (measured after the task) to a neutral baseline (50 %). Pearson correlation analyses were performed to test the relationship between participants’ recollection of the instances they received positive feedback and their level of social anxiety. To explore the possible differences in the reaction times (RT) for indicating positive and negative expectations a paired-sample *t*-test was performed comparing the RTs for participants’ acceptance (“YES”) and rejection (“NO”) expectancies during the SJP. Additional exploratory correlational analyses and *t*-tests were performed to investigate the relationship between pubertal development, and age, and expectancies and recollection of positive peer feedback. Bonferroni corrections for multiple comparisons were applied for these post-hoc analyses where appropriate.

Task-related differences in the SPN at different scalp regions during anticipation were tested via 2 × 4 repeated measures ANOVA with anticipated feedback Valence (2 levels: acceptance vs. rejection) and Cluster (4 levels: frontal left, frontal right, posterior left, posterior right) as within-subject variables. To test whether the SPN was modulated by our variables of interest, social anxiety, puberty, and, for completeness, age-related differences; three separate repeated measures ANCOVA (Social Anxiety, Puberty, and Age as covariates of interest) were performed in addition to the within-subject variables (i.e., anticipated feedback Valence and Cluster). The interaction effects were decomposed by repeated measures ANCOVAs for different clusters with Social Anxiety and Puberty as covariates. Pearson correlation analyses were performed to describe the directionality of the significant relationships between adolescents’ pubertal development and social anxiety, and the SPN during anticipation of acceptance and rejection. Subsequently, to explore the possible multiplicative effects of social anxiety and pubertal status, an ANCOVA was carried out with Social Anxiety, Puberty, and their interaction term as covariates. Given that pubertal status and social anxiety differed for boys and girls in our sample, we tested the effects of Puberty and Social Anxiety in *post-hoc* analyses for each sex at the right frontal and left posterior clusters. Huynh-Feldt corrected estimates, with uncorrected degrees of freedom for transparency, are reported where the sphericity assumption is violated as indicated by the Mauchly’s test of sphericity.

## 3 Results

### 3.1 Participant characteristics

Participants’ age and their levels of pubertal development were significantly correlated such that older adolescents had more advanced pubertal development, *r* (106) = .63, *p* < .001. In terms of the level of pubertal development, girls (*M* = 3.38, *SD* = 0.61) in our sample showed overall more advanced pubertal stage than boys (*M* = 2.72, *SD* = 0.62), *t* (104) = −5.40, *p* < .001, *d* = −1.05, 95 % CI [−1.46, −0.64]. Boys and girls did not differ in terms of their chronological age, *t* (104) = −0.513, *p* = .609, *d* = −0.1, 95 % CI [−0.48, 0.28]. Results of an independent-samples *t*-test showed that boys (*M* =−0.21, *SD* = 0.99) in our sample were less socially anxious compared to girls (*M* = 0.23, *SD* = 0.98), *t* (104) = −2.30, *p* = .024, *d* = −0.45, 95 % CI [−0.83, −0.06]. No significant correlations were found between participants’ age and social anxiety, or between pubertal development and social anxiety levels, *p*s < .05.

### 3.2 Social evaluative feedback expectancies

Firstly, we hypothesized that participants would display an overall positive social evaluative feedback expectancy bias. As shown in Table 4, participant’s overall feedback expectancy ratings prior to the task did not differ from chance level (i.e., 55 on the scale from 10 to 100), *t* (105) = 1.78, *p* = .077, *d* = 0.17, 95 % CI [−0.02, 0.37]. Nor did we find a significant positive social evaluative expectancy bias regarding the on-task predictions when tested against chance level (50 %), *t* (105) = −1.42, *p* = .158, *d* = −0.14, 95 % CI [−0.33, 0.05].

**Table 4:** Mean values and standard deviation for the behavioral variables.

	Mean (SD)
Baseline acceptance expectancies	58.39 (19.57)
On-task acceptance expectations (%)	48.32 (12.11)
Post-task recall acceptance	50.38 (15.43)
Acceptance RT (ms)	1311.82 (286.81)
Rejection RT (ms)	1292.41 (251.38)

Secondly, we hypothesized that socially anxious participants would be more pessimistic about social-evaluative feedback expectancies. We found no significant correlation between participants’ baseline expectancies to be liked by peers and social anxiety, *r* (106) = −.15, *p* = .135. Participants’ level of social anxiety was not significantly correlated with their trial-by-trial expectancies to be liked by peers during the SJP either, *r* (106) = −.04, *p* = .685. Neither age nor participant sex was related to baseline or trial-by-trial expectancies to be liked or with the amount of positive feedback recalled, *p*s > .05.

Thirdly, we hypothesized to find a negative social-evaluative feedback recall bias in socially anxious participants. Overall, participants showed a negative recall bias, *t* (104) =-3.07, *p* = .003, *d* = −0.30, 95 % CI [−0.49, −0.10], which showed that participants reported to have received less positive feedback (*M* = 50.38, *SD* = 15.43) than was actually presented to them (55 on a scale 10–100). The amount of recalled positive feedback from peers was not significantly correlated with the level of social anxiety, *r* (106) = −.13, *p* = .196.

Exploratively, we examined the reaction time (RT) data associated with participants’ predictions regarding the social evaluative outcome. Paired-sample *t-*test results showed that RTs for participants to indicate their expectancies to be liked (*M* = 1311.82 ms, *SD* = 286.81) or disliked (*M* = 1292.41 ms, *SD* = 251.38) by peers during the SJP did not differ significantly, *t* (105) = 1.32, *p* = .189, *d* = 0.13, 95 % CI [−0.06, 0.32]. Age, pubertal development, and social anxiety were not significantly associated with the RTs in either condition. However, boys (*M* = 1348.73 ms, *SD* = 261.88) compared with girls (*M* = 1229.34 ms, *SD* = 225.28) were slower to indicate their rejection feedback expectancy, *t* (104) = 1.43, *p* =.014, *d* = 0.49, 95 % CI [0.10, 0.87]. This difference was marginally significant when adolescents indicated their expectations to be accepted where girls (*M* = 1255.28 ms, *SD* = 263) were again quicker to respond than boys (*M* = 1362.31 ms, *SD* = 299.88), *t* (104) = 1.94, *p* =.055, *d* = 0.38, 95 % CI [−0.01, 0.76].

### 3.3 Stimulus preceding negativity (SPN)

For the SPN, we predicted that SPN amplitudes would correlate positively with self-reported levels of social anxiety, and there would be developmental differences in the SPN amplitudes which we investigated in relation to age and pubertal development.

**Fig. 3:**
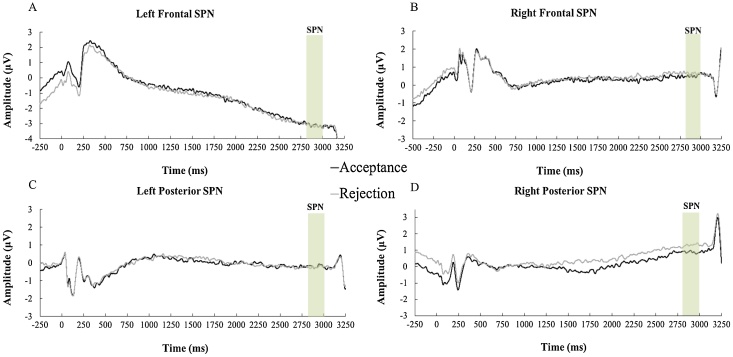
Grand averages for each condition at left frontal (A), right frontal (B), left posterior (C), and right posterior (D) sites.

First, we ran the main model with Valence (acceptance vs rejection feedback predictions) and Cluster (left frontal, right frontal, left posterior and right posterior) as within-subject factors. This model yielded a main effect for Cluster, showing that the SPN was largest at the left frontal cluster (see Fig. 3 and Supplemental Table S2), *F* (3, 315) = 71.31, *p* < .001, *η_p_^2^* = .40, 1 – *β* = 1. The main effect of Valence and the Valence x Cluster interaction were not significant (*p*s < .05). Subsequently, we separately report the effects of the covariates (i.e., Social Anxiety, Puberty, Age) within this main model.

**Fig. 4:**
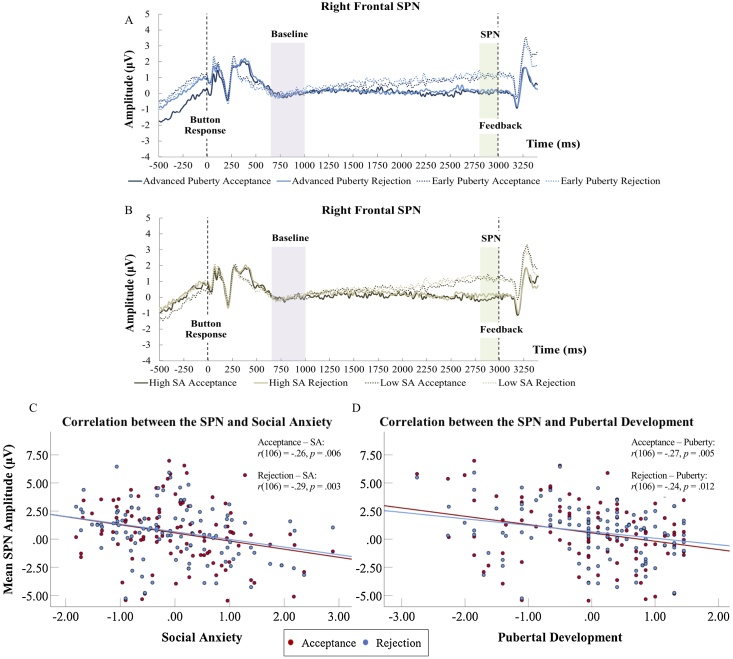
Graph showing the right frontal waveforms and SPN amplitudes during acceptance and rejection anticipation for advanced and early puberty (A) and high and low social anxiety (SA) groups (B) formed using median split. The median split method was not used in the analyses, but only for illustrative purposes to display the effects of puberty and social anxiety on the SPN waveforms. Negative values are plotted downwards. Associations between SPN amplitude (right frontal) and the level of social anxiety (C), and pubertal development (D). r refers to Pearson correlations.

To test the hypothesis that social anxiety would be positively related to the SPN, we ran the main model with Social Anxiety as covariate. We observed a significant interaction of Cluster and Social Anxiety, *F* (3, 312) = 4.57, *p* = .007, *η_p_^2^* = .04, 1 – *β* = 0.84 (see Table 5). Decomposition of this interaction showed a main effect of Social Anxiety at the right frontal, *F* (1, 104) = 10.17, *p* = .002, *η_p_^2^* = .09,1 – *β* = 0.89, and left posterior clusters, *F* (1, 104) = 6.91, *p* = .010, *η_p_^2^* = .06,1 – *β* = 0.74 (Supplemental Table S4). Next, we conducted post-hoc correlation analyses for Social Anxiety and the SPN for the right frontal and left posterior clusters to examine the directionality of the effects. We set alpha with Bonferroni correction at .0125 for four tests (two right frontal, two left posterior). Pearson correlations indicated that the right frontal SPN amplitude during both acceptance anticipation, *r* (106) = −.26, *p* = .006, and rejection anticipation, *r* (106) = −.29, *p* = .003, were significantly larger (more negative) for adolescents with higher levels of social anxiety (see Fig. 4). The correlation between the left posterior SPN amplitude (more positive) during rejection anticipation and social anxiety was also significant, *r* (106) = .25, *p* = .009. The correlation between the SPN during acceptance anticipation and social anxiety did not reach statistical significance, *r* (106) = .19, *p* = .058. These results are depicted in Fig. 4.

**Table 5:** The social anxiety, puberty, and age effects added in separate models.

Effect	df	Mean Square	F	*η_p_^2^*
Valence	1,104	2.88	2.00	.019
SA	1,104	11.64	2.61	.024
Cluster	3,312	915.20	73.74***	.415
Valence X SA	1,104	0.36	0.25	.002
Cluster X SA	3,312	56.76	4.57*	.042
Valence X Cluster	3,312	3.31	1.18	.011
Valence X Cluster X SA	3,312	2.55	0.91	.009

Valence	1,104	2.88	2.00	.019
Puberty	1,104	3.54	0.78	.007
Cluster	3,312	920.21	75.27***	.420
Valence X Puberty	1,104	0.15	0.10	.000
Cluster X Puberty	3,312	83.51	6.83**	.062
Valence X Cluster	3,312	3.32	1.18	.011
Valence X Cluster X Puberty	3,312	2.17	0.77	.007

Valence	1,104	2.88	2.01	.019
Age	1,104	27.47	6.38*	.058
Cluster	3,312	910.18	69.45***	.409
Valence X Age	1,104	0.93	0.65	.006
Cluster X Age	3,312	24.55	1.94	.018
Valence X Cluster	3,312	3.29	1.18	.011
Valence X Cluster X Age	3,312	3.20	1.15	.011

**Note.** ***p < .001, **p < .005, *p<.05.

To test whether the SPN would increase as a function of age, we ran the main model with Age as covariate. Results revealed a showed a main effect of Age on the SPN, *F* (1, 104) = 6.38, *p* = .013, *η_p_^2^* = .06, 1 – *β* = 0.71, such that older adolescents showed smaller overall SPN amplitudes (Table 5; Supplemental Fig. S2). There were no interaction effects between any other variable and Age, *p*s < .05. Except for the main effect of cluster that was retained in these models, no other main effect was observed, all *ps* > .05.

Lastly, to test whether the SPN would increase as a function of puberty, we ran the main model with Puberty as covariate. We observed a significant Cluster by Puberty interaction, *F* (3, 312) = 6.83, *p* = .001, *η_p_^2^* = .06, 1 – *β* = 0.95 (see Table 5). Pubertal status was found to be significant at right frontal, *F* (1, 104) = 9.82, *p* = .002, *η_p_^2^* = .09, 1 – *β* = 0.87, and left posterior, *F* (1, 104) = 16.82, *p* < .001, *η_p_^2^* = .14, 1 – *β* = 0.98, clusters (see Supplemental Table S4). Then, we conducted post-hoc correlation analyses for Puberty and the SPN for the right frontal and left posterior clusters. We set alpha with Bonferroni correction at .0125 for four tests (two right frontal, two left posterior). The level of pubertal development was significantly correlated with the SPN amplitude during anticipation of acceptance feedback, *r* (106) = −.28, *p* = .004. Similarly, the SPN during anticipation of rejection feedback and puberty were significantly correlated, *r* (106) = −.26, *p* = .006. As shown in Fig. 4, adolescents with more advanced levels of puberty showed larger (more negative) SPN for feedback anticipation. At the left posterior cluster, the SPN during both acceptance, *r* (106) = .29, *p* = .002, and rejection, *r* (106) = .36, *p* < .001, anticipation was correlated negatively with puberty, showing a more positive amplitude as participants’ pubertal stage increased.

We further explored whether there were multiplicative effects of social anxiety and puberty in addition to the reported effects on the electrocortical responses during anticipation of social evaluative feedback using a model with both Puberty and Social Anxiety, and their interaction. Except for the similar Cluster X Social Anxiety, *F* (3, 306) = 3.78, *p* = .016, *η_p_^2^* = .04, 1 – *β* = 0.7, and Cluster X Puberty, *F* (3, 306) = 5.99, *p* = .001, *η_p_^2^* = .06, 1 – *β* = 0.93, interactions previously reported for the right frontal and left posterior clusters, there were no significant multiplicative effects when accounting for both social anxiety and pubertal development, *p*s <.05 (see Supplemental Tables S3 and S5).

Exploratively, given the sex differences in social anxiety and pubertal status in our sample, we ran post-hoc analyses with puberty and social anxiety scores at the clusters where the effects were observed for boys and girls (see Supplemental Table S6). The results of these analyses showed social anxiety effects on the SPN at the right frontal, *F* (1, 48) = 6.36, *p* = .015, *η_p_^2^* = .12,1 – *β* = 0.70, and left posterior clusters, *F* (1,48) = 8.98, *p* = .004, *η_p_^2^* = .08,1 – *β* = 0.84, in girls but not boys. The effects of puberty remained significant in boys and at the left posterior cluster only, *F* (1, 54) = 6.31, *p* = .015, *η_p_^2^* = .11,1 – *β* = 0.69 (Supplemental Fig. S3).

## 4 Discussion

The objectives of this study were to (1) investigate social anticipatory processes in healthy adolescents as reflected in neural responses (i.e., SPN) during the anticipation of acceptance and rejection feedback from peers, and (2) examine how these neural responses related to individual differences in social anxiety and pubertal development. In addition, this study examined adolescents’ behavioral responses when they were asked to indicate their expectations regarding the social evaluative feedback they would receive from unknown peers. Importantly, our findings demonstrate individual differences in the neural correlates of adolescents’ anticipatory feedback processing as a function of social anxiety level, pubertal developmental, and age.

Contrary to our expectations, adolescents did not show an overall positive social evaluative feedback expectancy bias, as defined by their baseline expectancies to be liked relative to chance level, prior to or during the task. Most studies reporting the positive expectancy or optimism bias were conducted with adult or young adult populations. Recently, it has been suggested that such biases serve as a protective buffer and may still be developing in adolescence (Rodman et al., 2017). Similarly, adolescents might be more prone to internalize negative peer feedback (Rodman et al., 2017) which could be more salient and difficult for them to disregard. The fact that we studied an adolescent sample might be the reason why we did not observe this bias. These findings are also in line with those reported by Gunther Moor et al. (2010) where young adults had more acceptance expectations compared with younger adolescents and children. Nonetheless, these explanations are speculative as we did not compare this group of adolescents to adults in the current study. In terms of social anxiety, we observed no association with negative feedback expectancy in this sample. The adolescents we recruited for this study were not determined based on their clinical diagnoses or an anxiety threshold. Future research might consider recruiting adolescents with more extreme levels of anxiety to elucidate the differences in their behaviors while predicting social evaluation.

We observed a negative memory bias for the overall sample of adolescents. This is in line with the findings from previous studies using the SJP in adult samples which showed that individuals recalled having received significantly more rejection feedback than the actual proportion of rejection provided (Kortink et al., 2018; van der Molen et al., 2018). However, our results did not provide support for an increased negative memory bias with higher levels of social anxiety. It is possible that the changes in negative recall bias in relation to social anxiety symptoms become more apparent when the memory for the feedback is tested not shortly after the events take place but following a longer delay, (for example, see Glazier and Alden, 2017). Additionally, our sample consisted of healthy adolescents, even though there were also a group of adolescents scoring above the recommended cutoffs on the self-report measures of social anxiety (Beidel et al., 1995; Carleton et al., 2011; Wood et al., 2002). Thus, it might be possible to observe these effects in clinical samples when the data are examined continuously (Glazier and Alden, 2019). Together, these findings show that, although not apparent in their expectancies to be liked before or during the task, adolescents remember fewer instances of being liked by peers following socially evaluative experiences indicating a negative memory bias.

At the neural level, we observed that adolescents with higher levels of social anxiety displayed heightened SPN activity during social feedback anticipation. This effect was found at the right frontal cluster, which corresponds with prior studies demonstrating frontal dominance of the SPN (Hackley et al., 2020; Kotani et al., 2015, 2009; Masaki et al., 2010). We observed that the right frontal SPN was more negative for the anticipation of acceptance versus rejection feedback among adolescents with higher social anxiety symptoms, but this effect was not significant. Further, the results of our post hoc analyses in girls and boys showed that the social anxiety effects on the SPN were significant for girls but not boys consistent with previous findings showing that girls have increased risk for affective problems in adolescence (Ladouceur et al., 2012; Pfeifer and Allen, 2020). A previous study utilizing the SJP with thirty-one female young adults found a significantly more negative left posterior SPN during acceptance relative to rejection anticipation, which was modulated by increased fear of negative evaluation (van der Molen et al., 2014). Differences related to the sample characteristics of our study (i.e., adolescents) and measures (i.e., social anxiety) may explain why we did not see similar effects. It is possible that adolescents with higher social anxiety do not yet differentiate between negative and positive social evaluative feedback during anticipation. Instead, they might show larger reactivity for the anticipation of social evaluative feedback regardless of the valence of the feedback they expect, as in fear of negative and positive evaluation components of social anxiety (Weeks et al., 2008; Weeks and Howell, 2012). It could also be the case that adolescents with increased levels of social anxiety seek acceptance even by peers they think will not like them, whereas adults may be more invested in finding out about the feedback of those who they believe will like them and write off those who they think will not like them. Overall, these results provide evidence for the relationship between social anxiety symptoms and the neural correlates of social anticipation in adolescence, particularly among females.

In terms of neural response and puberty, adolescents with more advanced pubertal development showed a more negative right frontal SPN and the opposite effect at the left posterior cluster (Kotani et al., 2015; Luck, 2005). Our post hoc analyses indicated that this effect was significant in boys but not girls which might be related to the girls having more advanced pubertal development and less within-group variance in pubertal developmental status (see Supplemental Fig. S3). The same results on the SPN were not observed for chronological age. However, we did see a negative association between chronological age and the overall SPN. Although adolescents’ age and pubertal maturation were correlated, these findings suggest that differences in puberty might relate differently to adolescents’ neural responses during anticipation of social evaluative feedback than age. These findings may corroborate the literature on the differential effects of puberty on behavioral and motivational changes that are especially relevant to social contexts (Crone and Dahl, 2012; Forbes and Dahl, 2010; Sumter et al., 2010). Adolescents’ pubertal status is associated with hormonal changes and physical maturation and is thought to be a more sensitive indicator of developmental changes in social and motivational processes and reorganization of the related brain circuits than age (Crone and Dahl, 2012; Forbes and Dahl, 2010; Wierenga et al., 2018). This might be because pubertal timing can be different for adolescents of same age. Thus, it is possible that the influx of hormones and related changes in the brain that depend on pubertal maturation influence the salience and motivational value of anticipated peer feedback. Although we have not examined the changes in adolescents’ hormones directly, the findings of this study suggest that adolescents who are more physiologically mature, as indicated by self-reported pubertal development, may show greater reactivity while anticipating social evaluative feedback from peers.

The expected effects related to individual differences in social anxiety and puberty in the current study were observed at the right scalp sites. Different from our results, van der Molen et al. (2014) previously found the SPN effects on the left posterior sites during the SJP in a sample of adults. Our sample did not include adults precluding us from directly comparing adults and adolescents and identifying possible developmental differences in the lateralization of the SPN. Although, there are mixed findings regarding localization of the SPN in other tasks, several studies suggest a right preponderance of the SPN which is thought to arise from the right insular reactivity (Brunia et al., 2011; Hackley et al., 2020; Kotani et al., 2015, 2009). The effects we found prominently for the right clusters might be, therefore, explained by the lateral localization of the SPN.

### 4.1 Limitations and future directions

One possible limitation is that, even though a unique developmental sample took part in the study, the cross-sectional nature of the study did not allow us to examine the neural and behavioral changes within each individual over time. Thus, future research should consider employing longitudinal designs to study pubertal development and how it relates to changes neural responses during social anticipation. Relatedly, we found an association between the anticipatory neural responses and social anxiety levels in adolescents. Following these findings, it would be important for future studies to investigate whether heightened anticipatory responses to social evaluative feedback indicates risk for social anxiety problems by employing longitudinal approaches.

Adolescents recruited for this study did not have a social anxiety disorder diagnosis. Although it has been shown that individuals with subclinical social anxiety display interpretation biases and social impairments (Loscalzo et al., 2018), investigating the neural responses during the anticipation of social evaluative feedback in adolescents with clinical SAD can provide further insight into potential maladaptive processes. Lastly, this study employed a widely used self-report measure of pubertal development; however, future studies should also consider conducting an assessment of changes in specific hormones in puberty.

### 4.2 Conclusions

To the best of our knowledge, this study is the first to investigate the neural correlates of anticipation of social evaluative feedback in adolescents using EEG in a relatively large sample. Our findings reveal the differences in adolescents’ anticipatory processing that are related to development and social anxiety during their interactions with peers. Given the adaptive value of making predictions in social contexts, understanding the changes in neural responses during anticipation of social evaluation in adolescence bear importance for uncovering the processes involved in normative social and emotional development. Moreover, this study provides evidence that there are differences in anticipatory neural responses to social evaluative feedback related to adolescents’ social anxiety levels, particularly in girls, which might be underlying the maladaptive anticipatory processing seen in problematic social anxiety.

## Funding information

This work was supported by Mind Life Institute, 1440 Foundation (MJC), T32 MH18268 (MJC and CJW), 10.13039/100000027National Institute on Alcohol Abuse and Alcoholism (NIAAA): AA015496 and National Institute on Drug Abuse (NIDA): DA 007238 (BB), and Natural Sciences and Engineering Research Council (NSERC) of Canada Postdoctoral Fellowship (SvN).

## Data availability statement

The data that support the findings of the study are available from the senior author (MJC) upon reasonable request.

## Declaration of Competing Interest

The authors declare that they have no known competing financial interests or personal relationships that could have appeared to influence the work reported in this paper.

## Footnotes

1 As per reviewer’s request, we conducted an *a priori* power analysis which indicted that we would need a sample of 78−109 participants to identify a large effect. The details of this analysis can be found in the supplemental material (p.15).
