# Supplementary material for: As they wait: Anticipatory neural response to evaluative peer feedback varies by pubertal status and social anxiety
Source: Dev Cogn Neurosci. 2021 Aug 12;51:101004. doi: 10.1016/j.dcn.2021.101004 (PMC8377527; doi:10.1016/j.dcn.2021.101004)
Supplement: Supplementary file 1 [file mmc1.docx]

**Supplemental Materials**

**Table S1. Reliability of stimulus-preceding negativity (SPN) during anticipatory conditions**

| **Region/Condition** | Cronbach’s Alpha | Number of Trials |
| --- | --- | --- |
| *Right frontal* |  |  |
| “Yes” Anticipation | .82 | 60 |
| “No” Anticipation | .82 | 60 |
|  |  |  |
| *Left frontal* |  |  |
| “Yes” Anticipation | .76 | 60 |
| “No” Anticipation | .77 | 60 |
|  |  |  |
| *Right parietal* |  |  |
| “Yes” Anticipation | .83 | 60 |
| “No” Anticipation | .82 | 60 |
|  |  |  |
| *Left parietal* |  |  |
| “Yes” Anticipation | .84 | 60 |
| “No” Anticipation | .85 | 60 |
|  |  |  |

**Table S2. The differences of condition and channel cluster on anticipatory neural activity**

| **Effect** | **df** | **Mean Square** | **F** | ***η_p_^2^*** |
| --- | --- | --- | --- | --- |
| Valence | 1,105 | 2.88 | 2.02 | .019 |
| Cluster | 3,315 | 918.20 | 71.31*** | .404 |
| Valence X Cluster | 3,315 | 3.34 | 1.18 | .011 |

**Note.** ****p*<.001, ***p*<.005, **p*<.05

**Table S3. The social anxiety, and puberty in the same model**

| **Effect** | **df** | **Mean Square** | **F** | ***η_p_^2^*** | |
| --- | --- | --- | --- | --- | --- |
| Valence | 1,102 | 2.28 | 1.59 | .015 |  |
| Cluster | 3,306 | 890.19 | 74.97*** | .424 |  |
| SA | 1,102 | 12.89 | 2.87 | .027 |  |
| Puberty | 1,102 | 4.96 | 1.10 | .011 |  |
| SA X Puberty | 1,102 | 0.01 | 0.00 | .00 |  |
| Valence X SA | 1,102 | 0.59 | 0.41 | .004 |  |
| Cluster X SA | 3,306 | 44.88 | 3.78* | .036 |  |
| Valence X Puberty | 1,102 | 0.10 | 0.07 | .00 |  |
| Cluster X Puberty | 3,306 | 71.16 | 5.99* | .036 |  |
| Valence X SA X Puberty | 1,102 | 3.03 | 2.12 | .020 |  |
| Cluster X SA X Puberty | 3,306 | 6.16 | 0.52 | .005 |  |
| Valence X Cluster | 3,306 | 2.63 | 0.95 | .009 |  |
| Valence X Cluster X SA | 3,306 | 2.36 | 0.85 | .008 |  |
| Valence X Cluster X Puberty | 3,306 | 2.19 | 0.79 | .008 |  |
| Valence X Cluster X SA X Puberty | 3,306 | 3.11 | 1.12 | .011 |  |

**Note.** ****p*<.001, ***p*<.005, **p*<.05

**Table S4. Social anxiety and puberty at different channel clusters to decompose the interaction**

|  |  | | **Left Frontal** | | | |  | **Right Frontal** | | | |  | **Left Posterior** | | | |  | **Right Posterior** | | | |
| --- | --- | --- | --- | --- | --- | --- | --- | --- | --- | --- | --- | --- | --- | --- | --- | --- | --- | --- | --- | --- | --- |
| **Effect** | | **df** | | **MS** | **F** | ***η_p_^2^*** |  | **df** | **MS** | **F** | ***η_p_^2^*** |  | **df** | **MS** | **F** | ***η_p_^2^*** |  | **df** | **MS** | **F** | ***η_p_^2^*** |
| Valence | | 1,104 | | 0.13 | 0.04 | .00 |  | 1,104 | 0.55 | 0.27 | .003 |  | 1,104 | 0.12 | 0.06 | .001 |  | 1,104 | 11.23 | 5.64* | .051 |
| SA | | 1,104 | | 5.95 | 0.43 | .004 |  | 1,104 | 103.40 | 10.17** | .089 |  | 1,104 | 42.90 | 6.91* | .062 |  | 1,104 | 0.59 | 0.11 | .001 |
| Valence X SA | | 1,104 | | 5.85 | 1.81 | .017 |  | 1,104 | 0.09 | 0.05 | .00 |  | 1,104 | 1.40 | 0.74 | .007 |  | 1,104 | 0.07 | 0.04 | .00 |
| Valence | | 1,104 | | 0.13 | 0.04 | .00 |  | 1,104 | 0.55 | 0.27 | .003 |  | 1,104 | 0.12 | 0.06 | .001 |  | 1,104 | 11.23 | 5.73* | .052 |
| Puberty | | 1,104 | | 0.05 | 0.00 | .00 |  | 1,104 | 100.17 | 9.82** | .086 |  | 1,104 | 95.87 | 16.82*** | .139 |  | 1,104 | 14.06 | 2.79 | .026 |
| Valence X Puberty | | 1,104 | | 0.01 | 0.00 | .00 |  | 1,104 | 0.84 | 0.41 | .004 |  | 1,104 | 1.65 | 0.87 | .008 |  | 1,104 | 3.49 | 1.78 | .017 |

**Note.** ****p*<.001, ***p*<.005, **p*<.05

**Table S5. Model with both social anxiety and puberty at different channel clusters to decompose the interaction**

|  |  | | **Left Frontal** | | | |  | **Right Frontal** | | | |  | **Left Posterior** | | | |  | **Right Posterior** | | | |
| --- | --- | --- | --- | --- | --- | --- | --- | --- | --- | --- | --- | --- | --- | --- | --- | --- | --- | --- | --- | --- | --- |
| **Effect** | | **df** | | **MS** | **F** | ***η_p_^2^*** |  | **df** | **MS** | **F** | ***η_p_^2^*** |  | **df** | **MS** | **F** | ***η_p_^2^*** |  | **df** | **MS** | **F** | ***η_p_^2^*** |
| Valence | | 1,102 | | 0.13 | 0.04 | .00 |  | 1,102 | 0.38 | 0.18 | .002 |  | 1,102 | 0.07 | 0.04 | .00 |  | 1,102 | 9.10 | 4.79* | .045 |
| SA | | 1,102 | | 4.71 | 0.34 | .003 |  | 1,102 | 87.98 | 9.24** | .083 |  | 1,102 | 30.71 | 5.59* | .052 |  | 1,102 | 1.38 | 0.27 | .003 |
| Puberty | | 1,102 | | 0.38 | 0.03 | .00 |  | 1,102 | 83.51 | 8.77** | .079 |  | 1,102 | 83.85 | 15.25*** | .13 |  | 1,102 | 14.59 | 2.85 | .027 |
| SA X Puberty | | 1,102 | | 10.56 | 0.76 | .007 |  | 1,102 | 4.27 | 0.45 | .004 |  | 1,102 | 0.33 | 0.06 | .001 |  | 1,102 | 0.19 | 0.04 | .00 |
| Valence X SA | | 1,102 | | 5.81 | 1.76 | .017 |  | 1,102 | 0.01 | 0.01 | .00 |  | 1,102 | 1.27 | 0.66 | .006 |  | 1,102 | 0.14 | 0.07 | .001 |
| Valence X Puberty | | 1,102 | | 0.02 | 0.01 | .00 |  | 1,102 | 0.70 | 0.34 | .003 |  | 1,102 | 1.46 | 0.76 | .007 |  | 1,102 | 3.96 | 2.09 | .020 |
| Valence X SA X Puberty | | 1,102 | | 0.00 | 0.00 | .00 |  | 1,102 | 1.34 | 0.64 | .006 |  | 1,102 | 0.61 | 0.32 | .003 |  | 1,102 | 9.81 | 5.16* | .048 |

**Note.** ****p*<.001, ***p*<.005, **p*<.05

**Table S6. Separate models for social anxiety and puberty at right frontal and left posterior cluster in boys and girls**

|  |  | | **Boys** | | | | | | | | |  | **Girls** | | | | | | | | |
| --- | --- | --- | --- | --- | --- | --- | --- | --- | --- | --- | --- | --- | --- | --- | --- | --- | --- | --- | --- | --- | --- |
|  |  | | **Right Frontal** | | | |  | **Left Posterior** | | | |  | **Right Frontal** | | | |  | **Left Posterior** | | | |
| **Effect** | | **df** | | **MS** | **F** | ***η_p_^2^*** |  | **df** | **MS** | **F** | ***η_p_^2^*** |  | **df** | **MS** | **F** | ***η_p_^2^*** |  | **df** | **MS** | **F** | ***η_p_^2^*** |
| Valence | | 1,54 | | .00 | 0.00 | .00 |  | 1,54 | 0.11 | 0.06 | .001 |  | 1,48 | 0.15 | 0.09 | .002 |  | 1,48 | 0.58 | 0.31* | .006 |
| SA | | 1,54 | | 18.29 | 1.87 | .034 |  | 1,54 | 1.10 | 0.15 | .003 |  | 1,48 | 64.25 | 6.36* | .117 |  | 1,48 | 34.42 | 8.98** | .158 |
| Valence X SA | | 1,54 | | 1.54 | 0.66 | .012 |  | 1,54 | 6.99 | 3.87 | .067 |  | 1,48 | 2.74 | 1.56 | .031 |  | 1,48 | 1.86 | 0.99 | .020 |
|  | |  | |  |  |  |  |  |  |  |  |  |  |  |  |  |  |  |  |  |  |
| Valence | | 1,54 | | 1.32 | 0.58 | .011 |  | 1,54 | 0.17 | 0.09 | .002 |  | 1,48 | 1.43 | 0.80 | .016 |  | 1,48 | 0.31 | 0.02 | .00 |
| Puberty | | 1,54 | | 21.43 | 2.21 | .039 |  | 1,54 | 41.41 | 6.31* | .105 |  | 1,48 | 28.55 | 2.63 | .052 |  | 1,48 | 6.88 | 1.56 | .031 |
| Valence X Puberty | | 1,54 | | 4.24 | 1.86 | .033 |  | 1,54 | 0.78 | 0.41 | .007 |  | 1,48 | 1.03 | 0.57 | .012 |  | 1,48 | 0.20 | 0.10 | .002 |

**Note.** ****p*<.001, ***p*<.005, **p*<.05

**Supplemental Figure S1.**

A

B

Figure S1. Topo plots showing -200ms before feedback presentation for the (A) acceptance (“YES”) and (B) rejection (“NO”) anticipation.

**Supplemental Figure S2.**

Figure S2. Associations between chronological age (standardized) and overall SPN amplitude


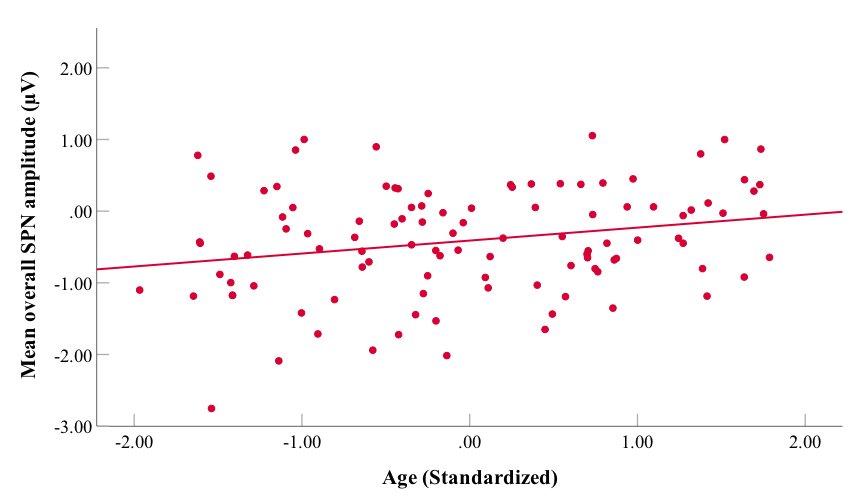


**Supplemental Figure S3.**


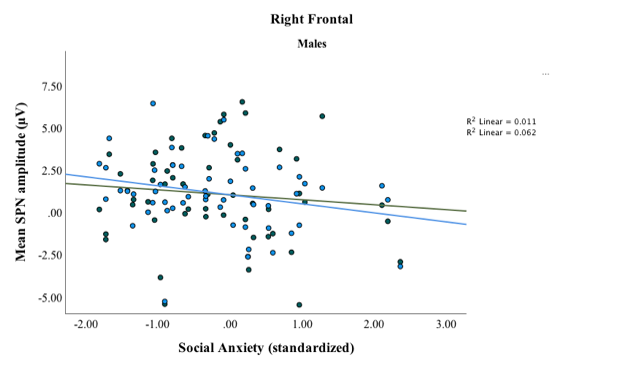


A1


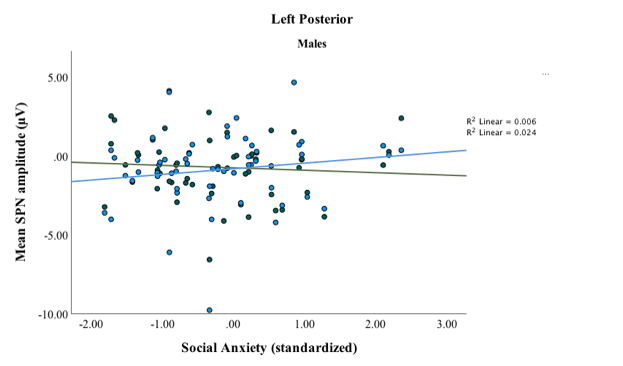


A2


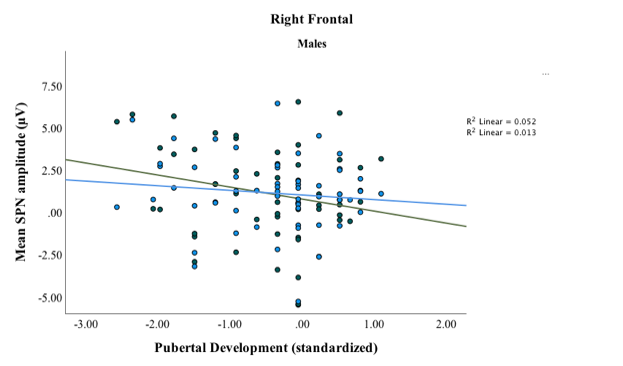

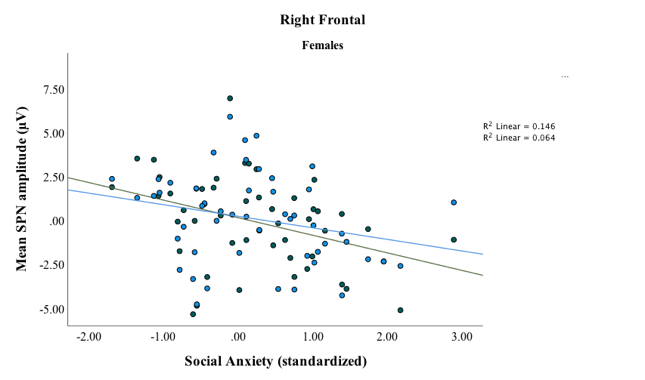


B1


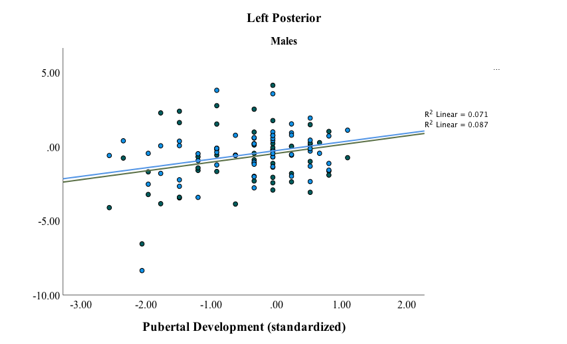

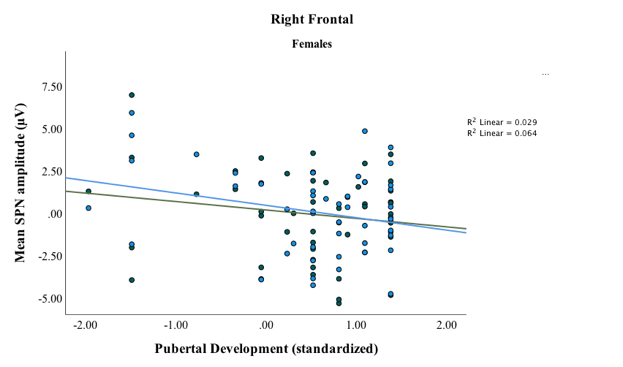


A4

*

B3

*

*


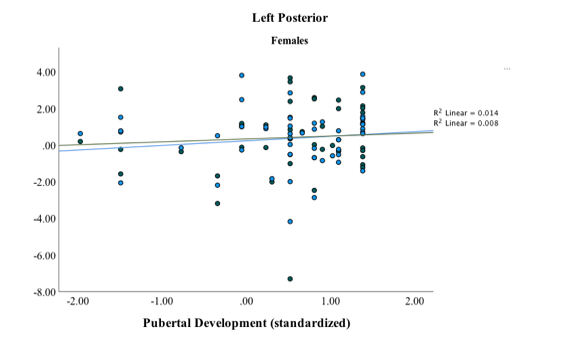

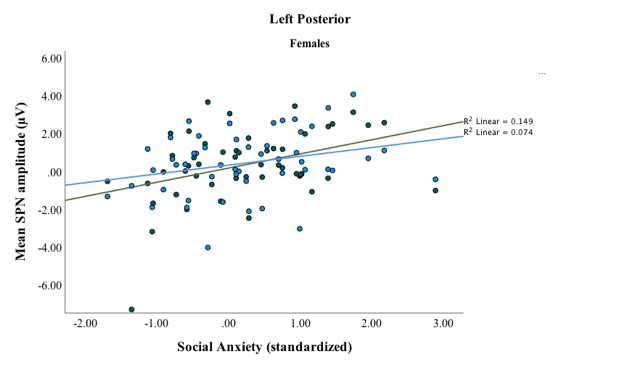


B4

A3

B2

Figure S3. Upper panel (A) shows males, lower panel (B) shows females. A1 and A3 shows how social anxiety and pubertal development relates to the SPN at the right frontal cluster in males respectively. A2 and A4 shows how social anxiety and pubertal development relates to the SPN at the left posterior cluster in males respectively. B1 and B3 shows how social anxiety and pubertal development relates to the SPN at the right frontal cluster in females respectively. B2 and B4 shows how social anxiety and pubertal development relates to the SPN at the left posterior cluster in females respectively. The blue data points and lines represent the acceptance (“YES”) and dark green data points and lines represent the rejection (“NO”) anticipation conditions. *p < .05

**Overall Study Protocol Measures**

Main study recruitment procedures are described in the manuscript. Below are the questionnaires administered to youth and a parent (where indicated) and the tasks administered along with concurrent EEG and electrocardiogram assessment.

**Measures**

**Demographic Information.** Each youth participant’s primary caregiver reported on family demographic information including their relationship with the child (biological, adoptive, etc.), family configuration, their ethnicity, marital status, partner’s ethnicity, their and their partner’s educational status, income, child’s ethnicity, grade level and if grade repeated.

**Children’s Depression Inventory (CDI)** (Kovacs, 1992) is a self-report scale or a parent report scale (CDI-P) that measures cognitive, behavioral, and neurovegetative signs of depression in children and adolescents aged 7 to 17. The scale consists of 27 items adapted from the Beck Depression Inventory for adults (Beck & Beamesderfer, 1974). The respondent chooses the statement that best describes him or her over the past two weeks (e.g., “I feel like crying every day/many days/once in a while”). The CDI total score is calculated by summing all the responses, with higher scores corresponding to greater severity of depressive symptoms. Both the CDI and the CDI-P were administered.

**The Pubertal Development Scale** (**PDS**; Carskadon & Acebo, 1993) is a scale measuring children’s pubertal status without pictorial representations or interviews. It has five items rating physical development on a 4-point scale. An overall maturation score is calculated as the total sum of all items. Youth and parent versions were administered.

**Multidimensional Anxiety Scale for Children (MASC)** is a 39-item (4-point Likert ranging 0-3) scale that measures different anxiety dimensions such as physical symptoms, harm avoidance, social anxiety, and separation anxiety in children and adolescents (March et al., 1997). Both youth self-report and parent report measures were administered.

**Child Avoidance Measure–Self Report (CAMS)** and Parent Report (CAMP) (Whiteside, Gryczkowski, Ale, Brown-Jacobsen, & McCarthy, 2013) (CAMS) were developed to be single-factor s measures of a child’s tendency to avoid stimuli that elicit anxiety, fear, or worry. The questionnaire presented a stem statement (When I (or my child) feel(s) scared or worried about something…) and items were written to have adequate breadth of content coverage for approaches to avoiding anxiety provoking stimuli, including passive avoidance, active refusal, delay, and expressing anger. Youth provided their self-report and one parent rated their child.

**Social Phobia and Anxiety Inventory for Children (SPAI-C)** measures social anxiety in children through self-reports on a set of 26 items (3-point Likert ranging 0-2) related to assertiveness, social encounters, and public performance (SPAI-C; Beidel et al., 1995). Youth provided self-report on the SPAI-C.

**Brief Fear of Negative Evaluation (bFNE)** is a commonly used measure of social anxiety and consists of 12 items (5-point Likert scale ranging 0-5) related to worries about and fears of being negatively evaluated (bFNE; Carleton et al., 2006). Youth provided self-report on the SPAI-C.

A**voidance and Fusion Questionnaire for Youth (AFQ-Y**; Greco et al., 2008). The AFQ-Y is a 17-item youth questionnaire of cognitive fusion and experiential avoidance for children and adolescents (Greco et al., 2008; Venta, Sharp, & Hart, 2012). Higher scores on the AFQ-Y are associated with increased anxiety (Venta et al., 2012) and have been found to improve (decrease) following mindfulness treatment in adolescents (Tan & Martin, 2014).

**Child Adolescent Perfectionism Scale (CAPS**; Flett et al., 2016) measures the two dimensions “self-oriented perfectionism” (SOP, 12 items) and “socially prescribed perfectionism” (SPP, 10 items). SOP indicates excessively high personal standards and a need to fulfill them, whereas SPP imply the conviction that other people require perfection from oneself. The items are rated on a five-point Likert scale from false (1), neutral (3) to very true (5). Youth provided their self-report.

**Childhood Narcissism Scale (CNS;** Thomaes et al., 2008) was used to assess the children’s narcissism traits. It is a unidimensional measure including 10 items (e.g., “I think it’s important to stand out”) scored on a Likert-type scale ranging from 0 (not at all true) to 3 (completely true). Higher scores are indicative of higher levels of narcissism. Youth provided their self-report.

**Adapted Short Michigan Alcoholism Screening Test (ASMAST**) for Fathers (F-SMAST) and Mothers (M-SMAST) (Cummings & Griffin, 1999; Hodgins & Shimp, 1995), the F-SMAST and M SMAST, are 13-item questionnaires that assess an individual’s father’s (F-SMAST) and mother’s (M-SMAST) lifetime alcohol abuse.

**Self-Compassion Inventory for Youth** is a sixteen item self-report scale that assesses two aspects of self-compassion coping, with two subscales, self-compassionate coping (8 items, When I’m upset, I think about the good parts of myself...; When..., I remind myself that no one can be good at everything. ) and self-punitive coping (8 items, When I’m sad or upset I start to notice all that is wrong with my life; When I have bad or difficult feelings I think I must be a bad person.). This scale is in development. A copy of the scale is available on request from the senior author.

**Ostracism and Connection Scale for Youth (OSCY)** is a twenty-five-item trait perception self-report scale that assesses ostracism by (12 items) and acceptance by peers (13 items). Ostracism items include, “When I am with other people they leave me out.”; “People give me the silent treatment (don’t speak to me on purpose).” Acceptance items include: “People invite me to do things with them outside of school.”; “I have a friend I can talk with when I feel upset.” A copy of the scale is available on request from the senior author.

**Study Tasks**

This study consisted of a resting electroencephalogram (EEG) and four tasks (self-referential encoding task, (SRET); Flanker Task; Social Judgment Paradigm (SJP), Cyberball). EEG was collected along with concurrent electrocardiogram (ECG).

The **self-referential encoding task (SRET)** required participants to read a single-word adjective (e.g., “gloomy”, “joyful”) and indicate whether the word was self-descriptive. The 104-word list was adapted from the Oregon Self-Concept inventory (OSCI-II: (Tucker et al., 2003) and previous studies with the SRET (Auerbach et al., 2015; Waters & Tucker, 2016). Fifty-two positive and fifty-two negative words, representing desirable or non-desirable personality attributes, were balanced for number of letters and reading level. Participants completed three practice trials using affectively neutral words (e.g., “tall”, “boy”) prior to the start of the task. Data collection began when the participant confirmed that they understood the instructions and were ready to begin. In each trial, the stimulus was first presented for 500ms, followed by a fixation cross (1800ms), and then the question (“Does this word describe you?”), to which participants answered by quickly pressing buttons corresponding to “yes” or “no” on a button response box. If a response was not recorded after 2500ms, the text “too slow” was presented. The inter-trial interval, during which a fixation cross was displayed, was jittered between 1500 and 1700ms. Participants completed all 104 trials, which were presented in a pseudorandom order with no more than two stimuli of the same valence presented in succession. Stimuli were presented using E-Prime 2.0 (PST, Sharpsburg, PA, USA).

**Flanker Task.** Stimuli were presented on a 19-inch monitor (refresh rate of 60 Hz) using E-prime 2.0 software (Psychology Software Tools, Pittsburgh PA). The same trial timings were used for all trials (practice and main blocks). A black fixation cross was presented on the center of the screen for 500 milliseconds and immediately followed by a blank screen (~1860 ms). Next, the flanker stimulus, which was displayed until the participant made a response on each trial. After a response, the fixation cross for the next trial was presented, where the central target could face either left or right. The central target was flanked by two arrows on each side, either in the same direction as each other or different directions from the others. On congruent trials, the flankers faced the same direction as the target (e.g., <<<<<), and on incongruent trials the flankers faced the opposite direction as the target (e.g., >><>>). The participants were instructed to make a spatially congruent response as to the direction of the central target by pressing the “Z” key for a left response and the “M” key for a right response. The stimulus from each trial was selected randomly (with replacement) from the total set of four possible stimuli. The stimuli were constrained such that all stimuli occurred equally often in each block. Participants were asked to respond as quickly and as accurately as possible once the stimulus appeared.

**Social Judgment Paradigm.** The adapted version of the Social Judgment Paradigm (SJP) was employed in the study (Gunther Moor et al., 2010; Somerville et al., 2006; Van der Molen et al., 2014). A cover story was used in this task where participants were led to believe that they were participating in a study on first impressions. Two weeks prior to the testing day, portrait photographs were collected from the participants. The participants were told that a panel of peers would evaluate them based on their photographs by reporting that the peer either liked or disliked the participant. When participants came into the lab on testing day, approximately two weeks later, they received the instructions that they would be shown the photographs of the peers who evaluated them and were asked to indicate whether they thought each peer liked or disliked them. In reality, the participants were not evaluated by actual peers and the like/dislike feedback was generated by computer. Following the participants’ response, the fictitious peer feedback was presented in each trial in a pseudo-random order. Participants received acceptance feedback 50% of the time. Different combinations of participants’ expectancies and the feedback they were shown resulted in four experimental conditions upon receipt of feedback: expected acceptance, expected rejection, unexpected acceptance, and unexpected rejection. Given that the current study focused on the period after participants reported their expectations and right before they received feedback, there were two conditions for anticipation: anticipation of acceptance and anticipation of rejection from peers.

A diverse in-house set of adolescent stimuli collected at the Developmental Electrophysiology Lab was used in the task which consisted of 160 peer photographs with a neutral facial expression (50% female). Stimuli were presented on a 19-inch monitor with a refresh rate of 60 Hz using E-prime 2.0 software (Psychology Software Tools, Pittsburgh PA).

In each trial, a photograph of a peer was presented as a cue for a maximum duration of 3000 ms during which the participants were required to provide a response showing their expectancies. If they did not provide their response within this time-interval, they were presented with the feedback “too slow”. For each peer stimulus, participants indicated whether they expected to receive acceptance (“YES”) or rejection (“NO”) feedback from that particular peer by pressing one of the two buttons with their index fingers. The order of which buttons corresponded to acceptance and rejection expectancies was counterbalanced across participants. Following the participants’ response was a delay period for the fixed duration of 3000 ms which was used to study anticipation. The duration of feedback presentation was varied across conditions with the purpose of generating a condition-specific marker for the heart rate recordings which are not within the scope of the current report. Specifically, feedback for the “Yes – Yes” condition (expected acceptance) was presented for 300 ms, feedback for the “Yes – No” (unexpected rejection) was presented for 400 ms; and for “No – Yes” (unexpected acceptance) and “No – No” (expected rejection) the durations were 500 ms and 600 ms respectively. Feedback presentation was followed by a jittered intertrial interval (ITI) between 500 – 1000 ms where the participants were shown a fixation cross in the middle of the screen. There were 10 practice trials in the beginning of the task preceding the three experimental blocks containing 50 trials each. Following the task administration, participants filled out a number of self-report questionnaires. Debriefing was done through a letter provided after each session was completed.

**The Cyberball Social Exclusion Task.** Cyberball is a virtual ball-toss game in which a participant plays with two other players on a computer. Abruptly, the others exclude the participant, only throwing to one another. This exclusionary experience is distressing to participants, as per their self-reports of distress on a Need Threat Scale (described below) (Eisenberger, Lieberman, & Williams, 2003; Williams, 2007).

When the game began, the child's glove was at the bottom center of the screen; the gloves of the other two players, chosen by the computer, were to the left and right of the screen center. Pictures of the other “players” appeared above their names and respective gloves. Participants used their left and right index fingers on a response pad to throw left or right to the other players. The child was led to believe s/he would be playing with two other children over the internet. Then the child was told a picture was taken of them with a camera (focused on them) that the other players would see. The child then overheard one experimenter telling a second experimenter s/he would knock on the door (closed) when the other players were ready to play on the internet. Three to five minutes elapsed before the knock occurred.

Prior to beginning the experiment, the child’s gender and ethnicity were identified. Settings within the game ensured that the other players on the screen were of a similar age, ethnic appearance and gender (drawing on a bank of opponent pictures taken at the Child Study Center for use in research). At the outset of the game, the child saw an actual GoogleTM webpage, followed by a “Cyberball” web page, followed by a screen with a green status bar. Several other modifications were introduced to make the Cyberball game more engaging to children. The child chose from one of six different ball gloves to be his or her personal glove throughout the game. A female voice narrated instructions on the computer screen. From throw to throw, the ball traveled randomly along different paths (straight line, arc or sine wave); lifelike sound effects occurred as the ball traveled (swoosh) and landed in a glove. After the experiment, the child and parent were debriefed and informed that the other players were not real.

Our ERP version of Cyberball consisted of 155 trials across two blocks, a fair play block (108 trials) and then an exclusion block (47 trials). During the 108-trial fair play block, the cyber-players threw to the participant 36 times. Whether a ball was thrown to the participant during any one trial was pseudorandom and predetermined within a list such that the participant waited for either 0, 1, 2 or 3 throws by the other players before receiving the ball again (frequency 12, 12, 10 and 2, respectively). Cyber-players threw to one another and not to the participant 36 times (“not my turn” events). The participant threw back to the other “players” for the remaining 36 trials. Seamlessly, fair play folded into a 47-trial exclusion block. This block represented 96% exclusion. Of the 47 exclusion trials, the ball only came to the participant three times to maintain attention, once on trial fourteen, twenty-five and thirty-nine.

Immediately after the game, children completed the Need Threat Scale (van Beest & Williams, 2006), a reliable and valid 20-item ostracism distress measure (Masten et al., 2009; Sebastian, Viding, Williams, & Blakemore, 2009; van Beest & Williams, 2006) which has been related to fMRI BOLD signal in previous research (Eisenberger et al., 2003). A female voice stated each item and the child made his or her response to the item with a mouse. Once it was clear the child understood how to use the mouse, the experimenter left the room while the child completed the need threat assessment. The Need Threat Scale gauges feelings of distress along four dimensions (5 items each): belonging (“I felt rejected”), self-esteem (“I felt liked”), meaningful existence (“I felt invisible.”), control (“I felt powerful”), on a 5-point choice, from “Not at all” to “Extremely”. A majority of the research on the neural correlates of social exclusion relies on the sum of these four scales as an index of ostracism distress. For this scale, higher scores indicated greater distress.

**Power Analysis**

An *a priori* power analysis was conducted to determine an appropriate sample size to detect an effect during the social judgment paradigm (SJP) using a repeated measures ANOVA design. Past research examining the SJP through a similar design reported a Cohen’s *f*  of .40 (Van der Molen et al., 2014), and we anticipate a comparable effect in our study. The WebPower package (Zhang & Yuan, 2018) in R (R Core Team, 2017) was used to calculate the sample size needed to detect the magnitude of this effect. Number of measurements specific to the current study (e.g., number of EEG channels, experimental conditions) were included in the WebPower function used in the analysis. Results indicated that a sample of approximately 100 individuals would achieve a power of .80 at alpha set to .05 for between-subject effects, approximately 78 individuals for within-subject effects, and 109 for interaction effects. Overall, samples sizes between 90 and 110 reflect power estimates ranging from .86 to .92 for within-subject effects.

Figure S4. Statistical power curve showing the sample size on the x-axis and the power on the y-axis

**References**

Beidel, D. C., Turner, S. M., & Morris, T. L. (1995). A new inventory to assess childhood

social anxiety and phobia: The Social Phobia and Anxiety Inventory for Children.

*Psychological Assessment, 7*(1), 73–79. <https://doi.org/10.1037/1040-3590.7.1.73>

Carleton, R. N., Mccreary, D. R., Norton, P. J., & Asmundson, G. J. (2006). Brief fear of

negative evaluation scale-revised. *Depression and Anxiety, 23*, 297-303.

<https://doi.org/10.1002/da.20142>

Carskadon, M. A., & Acebo, C. (1993). A self-administered rating scale for pubertal

development. *Journal of Adolescent Health, 14*(3), 190-195.

https://doi.org/10.1016/1054-139X(93)90004-9

Cummings, S. J., & Griffin, J. A. (1999). Identifying offspring of problem-drinking parents:

comparison of five self-report measures. *Substance Use and Misuse, 34*(13), 1817-1836. doi:10.3109/10826089909039428

Eisenberger, N. I., Lieberman, M. D., & Williams, K. D. (2003). Does rejection hurt? An

FMRI study of social exclusion. *Science, 302*(5643), 290-292.

doi:10.1126/science.1089134

Flett, G. L., Hewitt, P. L., Besser, A., Su, C., Vaillancourt, T., Boucher, D., . . . Gale, O.

(2016). The Child–Adolescent Perfectionism Scale. *Journal of Psychoeducational*

*Assessment, 34*(7), 634-652. doi:10.1177/0734282916651381

Greco, L. A., Lambert, W., & Baer, R. A. (2008). Psychological inflexibility in childhood

and adolescence: development and evaluation of the Avoidance and Fusion

Questionnaire for Youth. *Psychological Assessment, 20*(2), 93-102.

doi:10.1037/1040-3590.20.2.93

Gunther Moor, B., Van Leijenhorst, L., Rombouts, S., Crone, E., & Van Der Molen, M.

(2010). Do you like me? Neural correlates of social evaluation and developmental

trajectories. *Social Neuroscience, 5*(5-6), 461-482.

<https://doi.org/10.1080/17470910903526155>

Hodgins, D. C., & Shimp, L. (1995). Identifying adult children of alcoholics: methodological

review and a comparison of the CAST-6 with other methods. *Addiction, 90*(2), 255-

267. doi:10.1046/j.1360-0443.1995.90225511.x

March, J., Parker, J., Sullivan, K., Stallings, P., & Conners, C. (1997). The multidimensional

anxiety scale for children (MASC): Factor Structure, reliability, and validity.

*Journal of the American Academy of Child & Adolescent Psychiatry, 36*(4), 554-565.

<https://doi.org/10.1097/00004583-199704000-00019>

Masten, C. L., Eisenberger, N. I., Borofsky, L. A., Pfeifer, J. H., McNealy, K., Mazziotta, J.

C., & Dapretto, M. (2009). Neural correlates of social exclusion during adolescence:

understanding the distress of peer rejection. *Social Cognitive and Affective*

*Neuroscience, 4*(2), 143-157. Retrieved from

<http://www.ncbi.nlm.nih.gov/entrez/query.fcgi?cmd=Retrieve&db=PubMed&dopt=Citation&list_uids=19470528>

R Core Team (2017). *R: A language and environment for statistical computing*. R Foundation

for Statistical Computing, Vienna, Austria. URL <https://www.R-project.org/>.

Sebastian, C., Viding, E., Williams, K. D., & Blakemore, S. J. (2009). Social brain

development and the affective consequences of ostracism in adolescence. *Brain and*

*Cognition, 72*(1), 134-145. Retrieved from <http://www.ncbi.nlm.nih.gov/entrez/query.fcgi?cmd=Retrieve&db=PubMed&dopt=Citation&list_uids=19628323>

Somerville, L. H., Heatherton, T. F., & Kelley, W. M. (2006). Anterior cingulate cortex

responds differentially to expectancy violation and social rejection. *Nature*

*Neuroscience, 9*(8), 1007–1008. https://doi.org/10.1038/nn1728

Tan, L., & Martin, G. (2014). Taming the adolescent mind: A randomised controlled trial

examining clinical efficacy of an adolescent mindfulness-based group programme.

*Child and Adolescent Mental Health*. Retrieved from

<http://www.scopus.com/inward/record.url?eid=2-s2.0-84894517354&partnerID=40&md5=f085298865ac57c71a42526bc9197fcb>

Thomaes, S., Stegge, H., Bushman, B. J., Olthof, T., & Denissen, J. (2008). Development and

validation of the childhood narcissism scale. *Journal of Personality Assessment, 90*(4), 382-391. doi:10.1080/00223890802108162

Van der Molen M.J.W., Poppelaars, E.S., Van Hartingsveldt C.T.A., Harrewijn, A., Gunther

Moor B., & Westenberg, P.M. (2014). Fear of negative evaluation modulates

electrocortical and behavioral responses when anticipating social evaluative feedback.

*Frontiers in Human Neuroscience, 7*(936), 1-12.

<https://doi.org/10.3389/fnhum.2013.00936>

van Beest, I., & Williams, K. D. (2006). When inclusion costs and ostracism pays, ostracism

still hurts. *Journal of Personality and Social Psychology, 91*(5), 918-928.

Venta, A., Sharp, C., & Hart, J. (2012). The relation between anxiety disorder and

experiential avoidance in inpatient adolescents. *Psychological Assessment, 24*(1),

doi:10.1037/a0025362 21895380

Whiteside, S. P., Gryczkowski, M., Ale, C. M., Brown-Jacobsen, A. M., & McCarthy, D. M.

(2013). Development of child- and parent-report measures of behavioral avoidance

related to childhood anxiety disorders. *Behavior Therapy, 44*(2), 325-337.

doi:10.1016/j.beth.2013.02.006

Williams, K. D. (2007). Ostracism. *Annual Review of Psychology, 58*, 425-452.

Zhang, Z., & Yuan, K.-H. (2018). *Practical Statistical Power Analysis Using Webpower and*

*R*  (Eds). Granger, IN: ISDSA Press.
